# Dry heating affects the multi-structures, physicochemical properties, and *in vitro* digestibility of blue highland barley starch

**DOI:** 10.3389/fnut.2023.1191391

**Published:** 2023-05-10

**Authors:** Shuang Liu, Hang Liu, Shanshan Gao, Shang Guo, Cheng Zhang

**Affiliations:** ^1^Shanxi Institute for Functional Food, Shanxi Agricultural University, Taiyuan, China; ^2^School of Food Science and Engineering, Hainan University, Haikou, China

**Keywords:** blue highland barley, starch modification, dry heat treatment, multi-structures, physicochemical properties, *in vitro* digestibility

## Abstract

As a physical method for starch modification, dry heating treatment (DHT) at high temperatures (150 and 180°C, respectively) was applied to blue highland barley (BH) starch with different durations (2 and 4 h). The effects on its multi-structures, physicochemical properties, and *in vitro* digestibility were investigated. The results showed that DHT had changed the morphology of BH starch, and the diffraction pattern remained an “A”-type crystalline structure. However, with an extension of DHT temperature and time, the amylose content, gelatinization temperature, enthalpy value, swelling power, and pasting viscosity of modified starches decreased, while the light transmittance, solubility, and water and oil absorption capacities increased. Additionally, compared with native starch, the content of rapidly digestible starch in modified samples increased after DHT, whereas those of slowly digestible starch and RS decreased. Based on these results, the conclusion could be drawn that DHT is an effective and green way to transform multi-structures, physicochemical properties, and *in vitro* digestibility of BH starch. This fundamental information might be meaningful to enrich the theoretical basis of physical modification on BH starch and extend the applications of BH in the food industry.

## 1. Introduction

Highland barley is a cultivar of hull-less barley (*Hordeum vulgare* L. var. *nudum* Hook. f) and is known as “Qingke” in Mandarin. In China, highland barley is mainly distributed on the Qinghai–Tibet Plateau (at a high altitude of an average of 4,000 m above sea level). It has been the essential staple food crop and economic crop relied on by Tibetans since the fifth century AD (1, 2). Because of the unique geographical conditions (seasonal drought, cold, hypoxia, and intense UV radiation), highland barley accumulates and possesses various nutrients and secondary metabolites, including β-glucan, protein, vitamins, proanthocyanidins, flavonols, and phenolic compounds (3), which are endowed with antioxidant, anti-tumorigenic, and antibacterial capacities for human health. Numerous research studies have elucidated that these phytochemicals equip multiple health benefits, which are associated with the potential to reduce the risk of certain diseases including diabetes (4), colonic cancer (5), hyperlipidemia (2), cardiovascular disease (6), and obesity (7). Therefore, over the past decade has gained increasing research interest. Among different varieties, colored highland barley, such as blue, black, and purple ones, is a precious germplasm resource. Particularly, the blue highland barley (BH), as the most widely cultivated and consumed in the Qinghai–Tibet Plateau region, contains 336.29–453.94 mg of gallic acid equivalents per 100 g of dry weight (DW) total phenolic acid with strong antioxidant activity (3).

Starch, the dominant component of highland barley grains, constitutes ~ 59–75% of kernel dry weight. The amylose content varies from 0 to 40% in different cultivars (8). Native highland barley starch shows typical bimodal granules with large amylose molecules and long amylopectin chains (9, 10). In comparison with other grain starches, highland barley starch has a higher pasting temperature and gelatinization index (8). The predicted glycemic index (pGI) of highland barley starch was ~ 39.4–47.5, belonging to a low-GI level and suitable for diabetics (11), which has gained extensive attention for its health-improving values. However, structural and physicochemical properties of starch significantly affect the eating quality and functional performance of highland barley, for instance, retrogradation, shear sensitivity, and thermal resistance; therefore, it is in urgent need to be modified for extending food and industrial applications. Studies into highland barley starch modification are relatively scarce. The advances in physical methods applied to the modification of highland barley starch have been reported on ANN (12), HMT (13), microwave irradiation (14), and roasting (15). The HMT could disintegrate the molecular chain, increase amylose content, and even promote the V-type structure formation of highland barley starch (13). The swelling power and gelatinization properties of highland barley starch were increased under microwave mainly by disrupting amylopectin clusters in the crystalline regions (14). Roasting decreased the swelling power, pasting viscosity, solubility, and gelatinization enthalpy of starches from highland barley while increasing the gelatinization temperature (15). However, more information about physical modification, in particular with BH starch, is imperative to be further explored.

Dry heating is a “green” and “eco-friendly” technology and has received long-standing attention due to its safety, simplicity, and low costs when compared with chemical technologies (16). Recently, dry heating treatment (DHT) has been broadly applied to starch modification for altering various properties without destroying the granular structure of different starches (17–19). This further improves the functional characteristics of starches as texturizing, filling, coating, and whitening agents for special applications. Considering that the effects of DHT on BH starch are rarely reported, in the present study, DHT was carried out on the BH starch to systematically unveil the effects on its structures and unique properties and then their relationship to the functionalities of starch. The results obtained from this study will enrich the theoretical basis of dry heating modification on blue highland barley starch, essentially filling up the blanks in our knowledge of BH starch modification, and promoting its utilization in the food industry.

## 2. Materials and methods

### 2.1. Materials

The BH grains (Ganqing 4#) with 14.6% moisture content were purchased from Qinghai Xinning Biotechnology Co., LTD. Standard amylopectin (10120; from maize), amylose (A0521; purity ≥ 70%, from potato), and porcine pancreatic α-amylase (A3176; 16 U/mg) were purchased from Sigma–Aldrich Chemical Co. (St. Louis, MO, USA). The amyloglucosidase from *Aspergillus niger* (100,000 U/g) was purchased from Nanjing Duly Biotech Co., Ltd (Nanjing, China). All the other chemicals were of analytical grades.

### 2.2. Starch isolation

The extraction of BH starch was performed according to the previously described literature by Zhao et al. (15). The obtained native BH starch (NBHS) was dried at 40°C in a constant temperature convection oven (DHG-9203A, Shanghai Jing Hong Laboratory Instrument Co., Ltd, Shanghai, China) until the moisture content reached < 10%. Then, it was ground into powder and screened through a 100 mesh sieve for further analysis.

### 2.3. DHT

The NBHS sample [40 g, dry basis (db)] was weighted into a heat-proof dish and distributed evenly in a thin layer (~1 mm). The dish was covered with aluminum foil to ensure no loss of material. Then, the sample dishes were heated in the oven at 150 and 180°C for 2 or 4 h, respectively (20). The corresponding DHT starches were named BH150-2, BH150-4, BH180-2, and BH180-4.

### 2.4. Scanning electron microscopy

A scanning electron microscope (SEM; S3400II, Hitachi Ltd., Tokyo, Japan) was used to observe the morphology of different samples. Different samples were adhered to a double-sided adhesive tape on a metal stub. The samples were coated with 20 nm of gold under vacuum and observed at an acceleration potential of 20 kV.

### 2.5. Confocal laser scanning microscopy

The native and modified samples were obtained by a CLSM according to the method reported by Gou et al. (21).

### 2.6. X-ray diffraction analysis

The XRD pattern of different samples was determined by an X-ray diffractometer (D/MAX 2,500 V, Rigaku Corporation, Japan). The scanning angle (2θ) was from 5° to 60° at a scanning rate of 4°/min under 40 kV at 30 mA current. The relative crystallinity (RC, %) was calculated with Jade 6.0 software (OriginLab Corporation, USA).

### 2.7. Fourier transform-infrared spectroscopy analysis

The FT-IR spectra of samples were determined by a Vertex 70 (Bruker, Germany) in a range of wavenumber from 400 to 4,000 cm^−1^.

### 2.8. Color analysis

Color measurement of samples was carried out with a Hunter Colorimeter (CS-820, Hangzhou CHNSpec Technology Co., Ltd, Hangzhou, China). It was equipped with an optical sensor based on L^*^, a^*^, and b^*^ color systems.

### 2.9. Amylose content and alkaline water retention

Amylose content (AMC) was determined by the iodine-binding procedure of Juliano et al. (22). The alkaline water retention (AWR) of samples was calculated with the method of Adebowale et al. (23).

### 2.10. Oil and water absorption capacities

The oil and water absorption capacities of samples were determined according to the method reported by Liu et al. (24). The sample (4 g, db), combined with 20 ml of water (or peanut oil), was transferred into a 50 ml centrifuge tube. The tubes were stirred every 5 min during a 30-min incubation at 30°C and centrifuged at 7,300 *g* for 15 min. The volume of decanted supernatant fluid was measured, and milliliters of water (or oil) retained per gram of different samples were calculated.

### 2.11. Solubility and swelling power

The solubility and SP of samples were determined following the method of Liu et al. (25). The sample (50 mg, db) was put into a dry centrifuge tube, weighed (W_1_), and mixed with 5 ml of distilled water. The tubes were incubated in a shaking water bath at 50, 60, 70, 80, and 90°C for 30 min, respectively, then cooled to room temperature and centrifuged at 657 *g* for 15 min. The supernatant was carefully decanted, and the resulting precipitate was weighed (W_2_). Solubility and SP per 100 g of sample on db were calculated using the following equations:


(1) $$\begin{array}{l} \text{Solubility}=\frac{\text{the weight of dried supernatant}}{\text{weight of sample}} \end{array}$$

(2) $$\begin{array}{l} \text{SP}=\;\frac{{\text{W}}_{\text{2}}-{\text{W}}_{\text{1}}}{\text{weight of sample}} \end{array}$$

### 2.12. Paste clarity

Paste clarity (light transmittance, LT) of samples was measured with the method reported by Zou et al. (26). The transmittance was determined against water blank at 650 nm by a spectrophotometer (UV-3100PC, Shanghai Mapada Instruments Co., Ltd. Shanghai, China,) at 0, 24, 48, and 72 h, respectively.

### 2.13. Differential scanning calorimetry

The gelatinization characteristics were assessed with a differential scanning calorimeter (Q2000, TA Instruments, New Castle, DE, United States). The sample (3 mg, db), mixed with 9 ml distilled water, was sealed and equilibrated at room temperature overnight. The sample pans were heated from 30 to 120°C at 10°C/min, and an empty pan was taken as control. Onset temperature (*T*_o_), peak temperature (*T*_p_), conclusion temperature (*T*_c_), and gelatinization enthalpy (Δ*H*) were determined.

### 2.14. Pasting properties

The starch sample (3 g, db) and distilled water (25 g) were dispersed evenly in an aluminum canister. A Rapid Visco Analyzer (RVA-4, Newport Scientific Co., Ltd., Warriewood, NSW, Australia) was used for the evaluation of pasting properties. The testing cycle was set at 50°C for 1 min, ramped to 95°C in 3.7 min, held at 95°C for 2.5 min, cooled to 50°C in 3.8 min, and finally held at 50°C for 2 min. Setback viscosity (SB), breakdown viscosity (BD), peak viscosity (FV), pasting temperature (PT), and final viscosity (FV) were measured.

### 2.15. *In vitro* digestibility

*In vitro* digestibility of different samples was performed according to previous research by Gao et al. (27). The content of rapid digestible starch (RDS), slow digestible starch (SDS), and resistant starch (RS) was calculated based on the hydrolysis curve.

### 2.16. Statistical analysis

Triplicate measurements were performed to obtain mean values and standard deviations. All data were statistically analyzed by one-way analysis of variance (ANOVA) by Duncan's multiple range test using SPSS version 19.0 software (SSPS Inc. Chicago, IL, USA). The least significant difference (LSD) test was performed to determine differences among the treatments. The principal component analysis (PCA) was conducted with Minitab version 17 (Minitab Inc., USA). Statistical significance was set at a level of *p* < 0.05.

## 3. Results and discussion

### 3.1. Morphological properties

**Figure 1:**
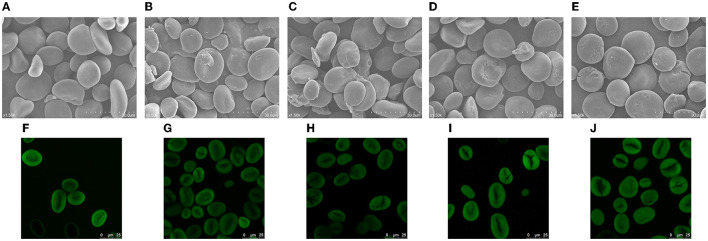
SEM (1,500 × ) and CLSM photographs of native and modified samples. **(A, F)** NBHS; **(B, G)** BH150-2; **(C, H)** BH150-4; **(D, I)** BH180-2; **(E, J)** BH180-4.

The SEM images of different starches are shown in Figure 1. The NBHS granules have irregular oval and polygonal shapes (Figure 1A). The surface of the granules was smooth without cavities or fissures. After DHT, a few potholes were found on the surface of BH150-2 (Figure 1B), while more cavities appeared on the surface of BH150-4 granules (Figure 1C). Compared with the preceding samples, the surface of BH180-2 granules was obviously eroded. The larger and deeper pits and holes were observed in the hilum (Figure 1D). In addition, some cracks and fissures developed on the surface of BH180-4 (Figure 1E). These morphological changes in BH starch were positively related to the temperature and duration of DHT. However, the treatment conditions used in this study were still insufficient to affect the integrity of NBHS. Water chestnut starch also showed a cracked surface following DHT (28), and similar observations on dioscorea and cassava starches modified by DHT were reported as well (19, 29).

A ring structure of amylose and amylopectin in starch granules grows outward from the hilum. The hilum contained substantial quantities of moisture, resulting in softening to be easily eroded by heating. It explained that the hilum of NBHS was more vulnerable to being sunken and damaged during DHT. The leaching of amylose and the reorganization of amylose–amylopectin possibly contributed to these morphological changes (26). Meanwhile, the high temperature would accelerate the movement of starch molecules during DHT and further lead to potholes or collapse as well (21). The generation of these potholes was also probably attributed to the transfer or rearrangement of central molecules in starch granules. These morphological alterations might facilitate the hydrolysis rate of starch by acid or enzymes.

The CLSM images of native and dry-heated samples are shown in Figure 1. The CLSM technique can characterize channels, growth rings, and pores of starch granules and also reveal the distribution of amylopectin and amylose. The hilum, as the central part of native starch, shows a uniform distribution of fluorescence areas with a clear growth ring (Figure 1F). Following DHT, the edges of starch granules and growth rings gradually blurred with ascending temperature and duration, and the bright area of fluorescence turned weaker (Figures 1G–J). The breakage and reassociation of amylose–amylopectin and amylose–amylose chains during DHT mainly contributed to the blurred granule edges (21). The weaker growth rings in modified samples might be attributed to the partial reorganizing of the amorphous region and the melting of crystals (17). Similar results had also been found on wheat, red adzuki bean, and mung bean starches in a range of studies (30–32).

### 3.2. XRD pattern and RC

**Figure 2:**
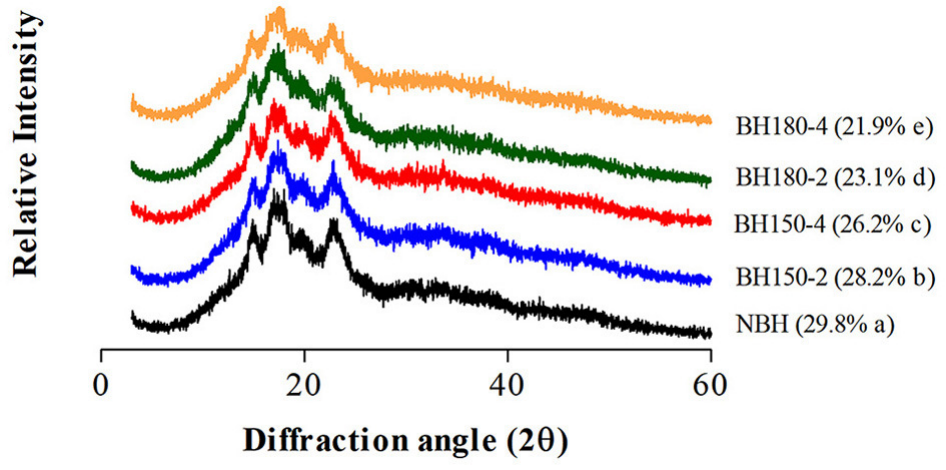
The XRD pattern and relative crystallinity (in parenthesis) of different samples.

The XRD pattern and RC of native and dry-heated starches are presented in Figure 2. The NHBS has a typical “A”-type crystalline pattern. The diffraction peaks were at 2θ angles of 15.20°, 17.36°, 18.00°, and 23.06°. Following DHT, similar diffraction angles of modified starches were found with the lower diffraction intensity, and this decrease in intensity was positively related to DHT temperature and time conditions. The DHT rarely influenced the original “A”-type crystalline pattern of NBHS, which demonstrated that changes in NBHS by DHT might primarily occur in the amorphous region (21). Our findings are consistent with previous studies where DHT was applied to rice starch under different treatment conditions (18).

Compared with the RC of NBHS (29.8%), the RC of modified starches was significantly decreased, ranging from 28.2 to 21.9% with the order of BH150-2 > BH150-4 > BH180-2 > BH180-4. The reduction in RC of modified starches was dependent on the temperature and duration of DHT, which might be attributed to the movement of double helical, partial gelatinization of starch granules, and even degradation of the crystalline region during DHT (17, 33). In addition, crystal size, amount of crystallinity in starch, and interaction between double helices during DHT may also engender the reduction in RC.

### 3.3. FT-IR spectroscopy analysis

**Figure 3:**
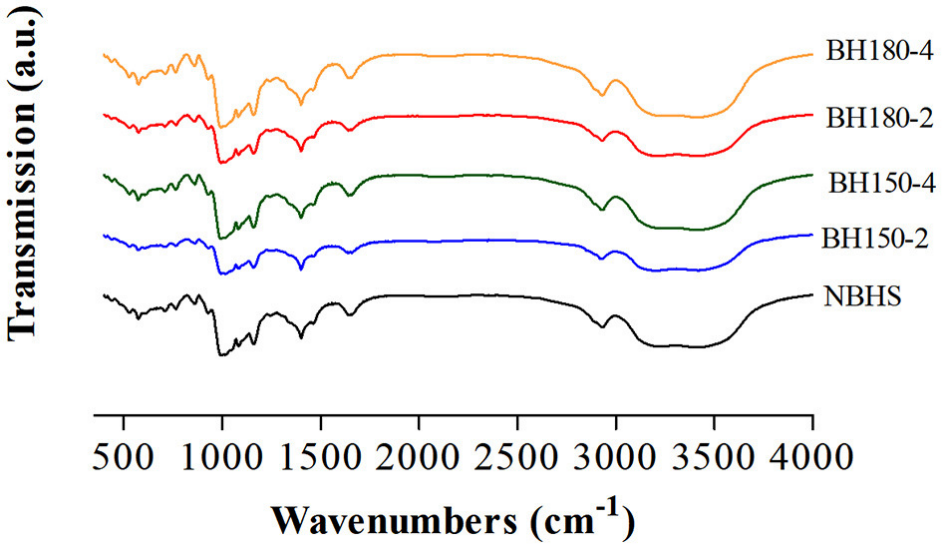
FT-IR spectra of native and modified starch samples.

The FT-IR spectroscopy represents the short-range ordered structure of starch (26). The FT-IR spectra of native and modified starches recorded from 400 to 4,000 cm^−1^ are shown in Figure 3, and the absorbance ratios of 1,047/1,022 cm^−1^ are shown in Table 1. Compared with NBHS, the modified starches had similar absorption peaks. It suggested neither new chemical groups had been formed nor the existing chemical groups had been destroyed during DHT in current cases.

**Table 1:** Color parameters and FT-IR 1,047/1,022 ratio of different samples.

**Samples**	*L* ^*^	*a* ^*^	*b* ^*^	**R_1, 047/1, 022_**
NBHS	95.8 ± 0.62a	0.14 ± 0.03d	1.4 ± 0.05e	1.09 ± 0.02a
BH150-2	94.4 ± 0.23b	0.20 ± 0.02c	3.9 ± 0.03d	1.07 ± 0.16b
BH150-4	93.7 ± 0.11c	0.33 ± 0.01b	6.1 ± 0.04c	1.06 ± 0.05c
BH180-2	91.6 ± 0.23d	0.36 ± 0.02b	10.3 ± 0.11b	1.04 ± 0.23d
BH180-4	89.3 ± 0.32e	1.05 ± 0.03a	13.6 ± 0.16a	1.03 ± 0.06e

Means of triplicate determination ± S.D with the different letter in a column within each property are significantly different (p < 0.05).

R_1, 047/1, 022_, the ratio of FT-IR value at wave 1,047 and 1,022.

Bands at 1,047 and 1,022 cm^−1^, respectively, represent the content of crystalline starch and amorphous starch. The absorbance ratio of 1,047/1,022 suggests the amount of ordered crystalline to amorphous domains in starch (21), associated with the crystal and amorphous lamellae density of the starch granule, arranged at a short length scale. The significant decrease in R_1, 047/1, 022_ for all DHT-modified starches compared to that of NBHS might be ascribed to the breakdown of the original hydrogen bonds, subsequently a dissociation in the double helices of the crystalline region (34). This was highly consistent with the XRD results in Section 3.2. The decreasing trend in the R_1, 047/1, 022_ value was positively associated with the raising of DHT temperature and duration. Therefore, the longer treatment time and higher temperature resulted in less of the short-range molecular order in the crystalline structure of modified starches. Similar results had also been reported when mung bean (32), red adzuki bean (31), and sweet potato starches (21) were treated by the dry heating process.

### 3.4. Color measurement

The influence of DHT on the color parameters of starches is shown in Table 1. The DHT significantly decreased the L^*^ value from 95.8 (NBHS) to 89.3 (BH180-4), while increased the a^*^ value from 0.14 (NBHS) to 1.05 (BH180-4) and b^*^ value from 1.4 (NBHS) to 13.6 (BH180-4).

The decrease in L^*^ value indicated that the NBHS turned darker after DHT and was pronouncedly followed by the temperature increase. The a^*^ value is relevant to redness characteristics. The increase in a^*^ value showed that the DHT led to more browning in modified starches, probably due to caramelization reactions (35). In addition, the significant rise in the b^*^ value evinced the greenness characteristics of NBHS, which is gradually increased by DHT. These change trends were positively dependent on the duration and temperature of DHT. Similar effects have been reported on DHT-modified potato, sweet potato, and taro starches as well as whole-grain barley (36).

### 3.5. AMC and AWR

The AMC of native and DHT-modified starches is shown in Table 2. Compared with NBHS, the AMC in DHT-modified starches significantly decreased by 10.09% (BH150-2), 12.52% (BH1150-4), 14.27% (BH180-2), and 18.59% (BH180-4), respectively. This decrease was positively related to the treatment temperature and duration. It might be ascribed to fragmenting into shorter chains under high-temperature conditions (37). Previous studies yielded similar results on DHT-modified chestnut and cassava starches (19, 38).

**Table 2:** AMC, AWR, and oil and water absorption capacity of different samples.

**Parameters**	**Samples**				
	**NBHS**	**BH150-2**	**BH150-4**	**BH180-2**	**BH180-4**
AMC (%)	24.40 ± 0.09a	14.31 ± 0.43b	11.88 ± 0.23c	10.13 ± 0.29d	5.81 ± 0.64e
AWR (g/g)	0.98 ± 0.04b	1.09 ± 0.06b	1.14 ± 0.09b	1.16 ± 0.09ab	1.34 ± 0.07a
Water absorption capacity (g/g)	1.86 ± 0.16b	1.88 ± 0.11b	1.91 ± 0.07b	2.09 ± 0.16a	2.32 ± 0.09a
Oil absorption capacity (g/g)	1.44 ± 0.04c	1.74 ± 0.04bc	1.94 ± 0.19ab	2.01 ± 0.26a	2.22 ± 0.15a

Means of triplicate determination ± S.D with the different letter in a row within each property are significantly different (p < 0.05).

Compared with NBHS, DHT-modified starches had a higher AWR level (Table 2). Among the modified samples, the AWR increased with DHT duration and temperature level. The BH180-4 had the highest value (1.34). The increased surface area (39) and the excessive dilution at high concentrations of starch (24) jointly accounted for the increase in AWR. Furthermore, the significantly increased water absorption capacity of DHT-modified starches might also play an important role to increase the AWR. Generally, the AWR of starch is positively correlated with cookie diameter, which could predict the expansion potential of a cookie during baking (40). Thus, the use of DHT-modified starches may result in a larger cookie diameter compared to that containing native starch. DHT-modified BH starch might be an alternative starch source integrated into cookie processing.

### 3.6. Oil and water absorption capacities

The oil and water absorption capacities of native and modified starches are shown in Table 2. Compared to NBHS, DHT-modified starches had higher oil and water absorption capacities. With increasing treatment temperature levels and duration, water absorption capacity significantly increased from 1.88 g/g (BH150-2) to 2.32 g/g (BH180-4), and oil absorption capacity increased from 1.74 g/g (BH150-2) to 2.22 g/g (BH180-4). Similar findings about cassava starch altered by DHT have also been reported (19).

The results showed that DHT made stronger hydroxyl–water molecule interactions in modified starches than those in NBHS. During DHT, some hydrogen bonds broke down between the amorphous and crystalline regions, which resulted in a slight expansion of the amorphous region. All these changes increased the hydrophilic tendency of starch molecules and contributed to a higher water absorption capacity in DHT-modified samples (24). The amorphous region has a higher water absorption capacity than the crystalline region in starch. Therefore, the results reflected the degree of amorphousness in DHT-modified starch granules (18), which was in accordance with the results in Section 3.2. The increase in oil absorption capacity after DHT treatment has been reported previously on cassava, maize, and rice starches (19, 41), showing that DHT-modified BH starch might be applied to energy-controlled food for the obese or health-conscious groups. Furthermore, Lorenz and Kulp (42) found that adding modified potato and wheat starches with higher water absorption capacity into dough improved the color, volume, and textural properties of bread. This suggested that DHT-modified BH starch could be applied to improve the sensory characteristics of bread products.

### 3.7. Solubility and swelling power

**Figure 4:**
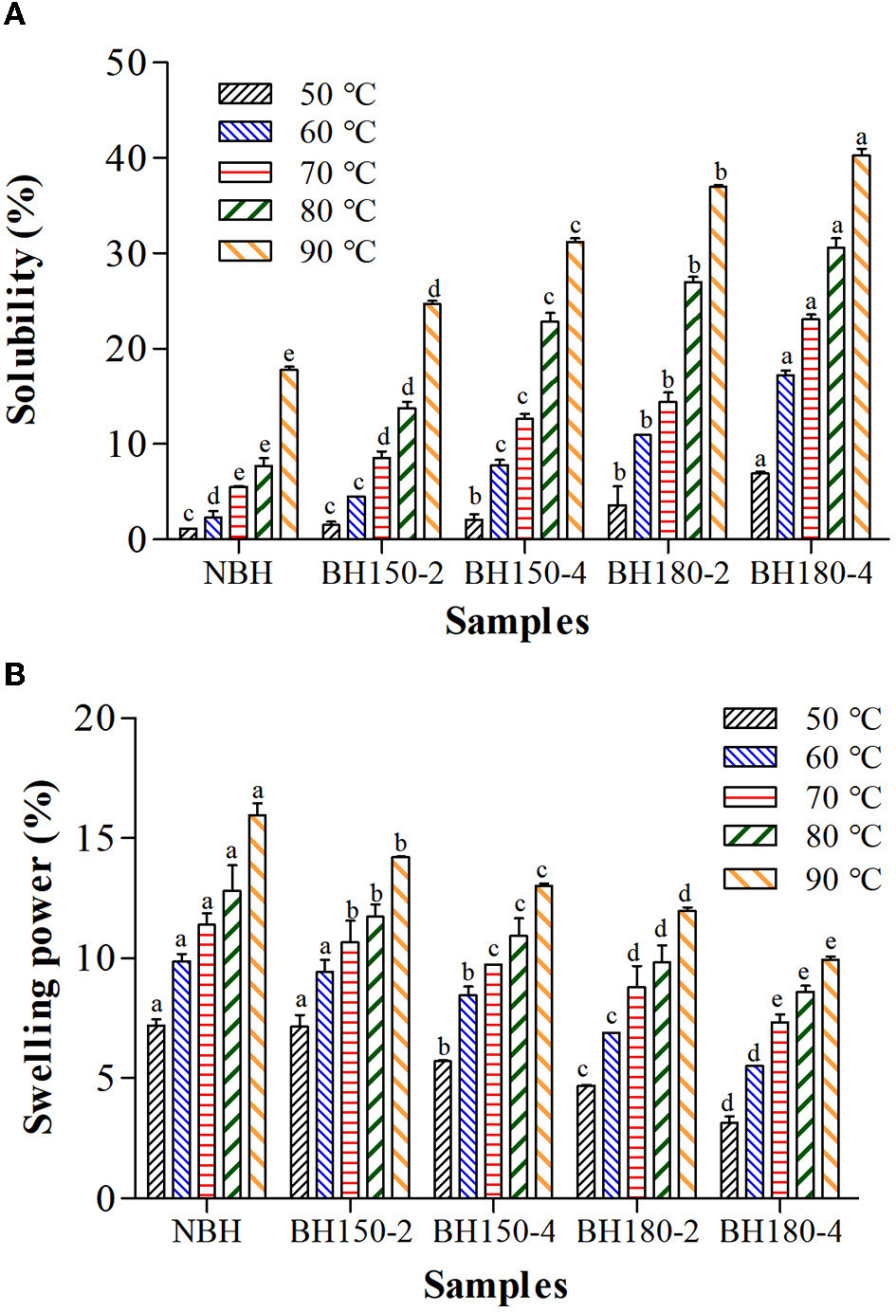
Solubility **(A)** and swelling power **(B)** of different samples. Bars bearing the same letter within the same temperature are not significantly different (*p* < 0.05).

The effect of DHT on solubility and SP of all starch samples is presented in Figure 4. For all samples, solubility and SP significantly increased with the treatment temperature raising, and the highest value was obtained at 90°C. The solubility of DHT-modified samples was significantly higher than that of NBHS (Figure 4A), while the SP remarkably decreased (Figure 4B). The solubility of starch is related to AMC. At high temperatures, partial double helices of NBHS began to unwind, fracturing into short molecules. It resulted in loosening and collapsing of the starch granule structure. Therefore, the increased solubility might be attributed to a high amount of short-chain amylose produced by DHT, which could diffuse out of granules and be easily dissolved (43). The interaction between starch molecules, double helices transition, and melting of starch crystallites might be other factors for the increase in the solubility of BH starch after DHT (31). The high temperature during DHT caused the complete migration of amylose from the surface of amylopectin crystals and increased the leaching of amylose, which resulted in higher solubility. A gradual decrease in the SP of starch was related to an increase in the amylose–amylopectin interaction due to the rearrangement of starch molecules from DHT (31). Furthermore, the SP decrease might also be triggered by hindered diffusion of amylopectin molecules after the rearrangement of crystalline regions. The decrease in SP was also promoted by the decreased AMC and the formation of the amylose–lipid complex during DHT (44).

### 3.8. Paste clarity

**Figure 5:**
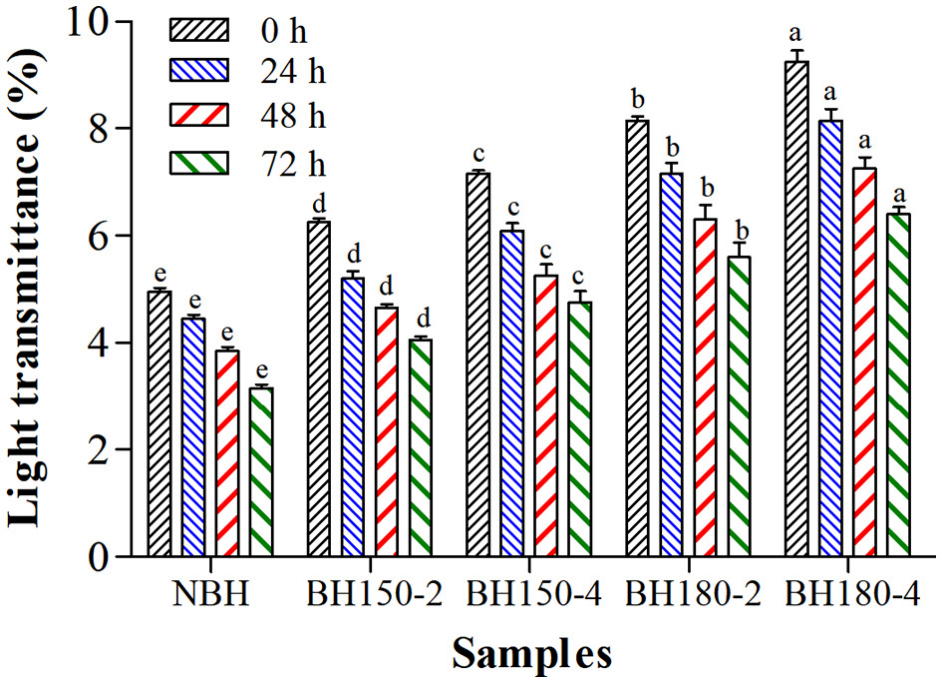
Light transmittance of different samples at different storage time. Bars bearing different letters within the same storage time are significantly different (*p* < 0.05).

In general, the LT reflects the clarity of starch paste, manifesting the retrogradation process (45). The LT of native and DHT-modified starches was determined at 0, 24, 48, and 72 h, as shown in Figure 5. As the temperature elevated and time prolonged, the LT of modified starches gradually increased compared with NBHS. These increases were attributed to the degradation or disintegration of starch chains induced by DHT (26). The increased LT of modified starches might also be due to the formation of new starch crystallites or recrystallization. Moreover, the LT is related to the solubility of starch, including greater solubility, less refraction, and more transparency of the paste. Similar results were also presented on DHT-modified waxy rice starch, normal rice starch, and waxy corn starch (26).

### 3.9. DSC

The gelatinization temperatures (T_o_, T_p_, and T_c_) and enthalpy (Δ*H*) of native and DHT-modified starches are presented in Table 3. Compared to NBHS, the T_o_, T_p_, T_c_, and Δ*H* of modified starches significantly decreased along with increased DHT temperature and duration. The gelatinization temperatures are indicative of the crystalline perfection in starch, where the more perfect crystallites show a higher T_o_ (46). After DHT, the significant reduction in T_c_, T_o_, and T_p_ of modified starches indicated that the inhomogeneity of starch double helix crystallites increased, which was in good agreement with the results from the XRD analysis. This shift was mainly due to the disruption of amylose. The double helices of amylopectin remained uncoiled and with a loose granular structure. Furthermore, the shortening of starch chains by the impaired binding forces in crystals might be another important factor for decreasing gelatinization temperatures and Δ*H* (16). The Δ*H*, a measure of double helix content and perfection of crystalline order in starch (47), decreased from 7.7 J/g (NBHS) to 6.5 J/g (BH180-4). This decrease was mainly attributed to the disruption of amylose and further destruction of the ordered structure during DHT (48). From these results, DHT affected the concentrated crystalline region and reduced the crystallinity of starch (49), which was consistent with XRD results as well. Similar results were also published on DHT-modified wheat, waxy corn, and high-amylose rice starches (18, 26, 30).

**Table 3:** Gelatinization characteristics of native and DHT samples.

**Samples**	**T_o_ (°C)**	**T_p_ (°C)**	**T_c_ (°C)**	**T_c_-T_o_ (°C)**	**Δ*H* (J/g)**
NBHS	55.8 ± 0.13a	59.6 ± 0.04a	65.6 ± 0.01a	9.8 ± 0.02a	7.7 ± 0.03a
BH150-2	54.6 ± 0.18b	57.5 ± 0.09b	64.3 ± 0.64b	9.7 ± 0.06a	7.5 ± 0.03b
BH150-4	53.4 ± 0.21b	56.6 ± 0.19c	62.9 ± 0.12c	9.5 ± 0.02a	7.4 ± 0.03c
BH180-2	52.6 ± 0.14c	55.6 ± 0.02d	61.9 ± 0.05d	9.3 ± 0.13a	7.3 ± 0.02c
BH180-4	51.5 ± 0.30d	54.5 ± 0.52e	60.5 ± 0.34d	9.0 ± 0.41a	6.5 ± 0.02d

Means of triplicate determination ± S.D with the same letter in a column within each parameter are not significantly different (p < 0.05).

T_o_, onset temperature; T_p_, peak temperature; T_c_, concluding temperature; T_c_-T_o_, gelatinization temperature range; ΔH, transition enthalpy.

### 3.10. Pasting properties

The viscosity properties of starch, resulting from the friction between the leaching of amylose and amylopectin, are functionally fundamental. The pasting parameters of native and modified samples are presented in Table 4. Compared to NBHS, the overall viscosities of DHT-modified samples significantly decreased with the increase in DHT temperature and duration and the PT. Zhang et al. found a similar pattern in wheat starch with different amylose contents after DHT (30).

**Table 4:** Pasting properties of different samples.

**Properties**	**Samples**				
	**NBHS**	**BH150-2**	**BH150-4**	**BH180-2**	**BH180-4**
PV (cP)	3,719 ± 18.4a	3,016.5 ± 21.9b	2,387 ± 8.5c	1,261.5 ± 3.5d	1,025.5 ± 12.0e
BD (cP)	2,120 ± 11.3a	1,781.5 ± 6.4b	1,461 ± 7.1c	490 ± 7.1d	276.5 ± 16.3e
SB (cP)	1,858 ± 21.2a	1,774.5 ± 7.7b	1,367 ± 26.8c	568 ± 4.2d	388 ± 15.5e
FV (cP)	3,457 ± 2.8a	3,009.5 ± 5.6b	2,293 ± 7.8c	1,339.5 ± 14.8d	1,137 ± 11.3b
PT (°C)	78.6 ± 0.1a	77.5 ± 0.6a	74.3 ± 0.1b	73.8 ± 0.1b	73.4 ± 0.1b
Pt (min)	5.2 ± 0.1a	5.1 ± 0.1ab	5.0 ± 0.1ab	4.9 ± 0.1ab	4.8 ± 0.2b

Means of triplicate determination ± S.D with the different letter in the row within each property are significantly different (p < 0.05).

“cP” is the rapid viscosity units.

PV, peak viscosity; BD, breakdown viscosity; FV, final viscosity; SB, setback viscosity; PT, pasting temperature; Pt, Peak time.

Generally, starch viscosity is mainly contingent on its source, granule size, crystal structure, and the ratio of amylose to amylopectin. The decrease in PV, SB, BD, and FV of modified starches might be attributed to the thermal degradation of amylose, amylopectin, and crystalline structure by DHT (26). The PV represents the maximum swelling value before starch granule disintegration and is associated with starch granular SP (43). The enhanced associations between starch chains and the improved intramolecular interaction and the reorganization of starch granules might also accelerate the decreased PV of modified starches (22). These results are consistent with those of SP analysis.

The FV reflects the stability of cold starch paste and the polymerization of starch molecules (50). It is related to starch gel hardness, retrogradation, and leached amylose content (51). The significant decrease in FV of DHT-modified samples was associated with changes in crystalline structure and short amylopectin. The SB represents the difference between FV and trough viscosity, showing the aging degree and stability of starch paste (50). Decreased SB demonstrated that DHT improved the stability of cold starch paste under shear force. Both decrease in FV and SB identified a lower tendency toward the retrogradation of modified starches. Moreover, the BD of modified samples was negatively related to DHT temperature and duration, and it indicated the enhancement in the heat resistance of starch. The decrease in both Pt and PT showed the gelatinization of modified starches became easier compared with that of NBHS.

### 3.11. *In vitro* digestibility

**Figure 6:**
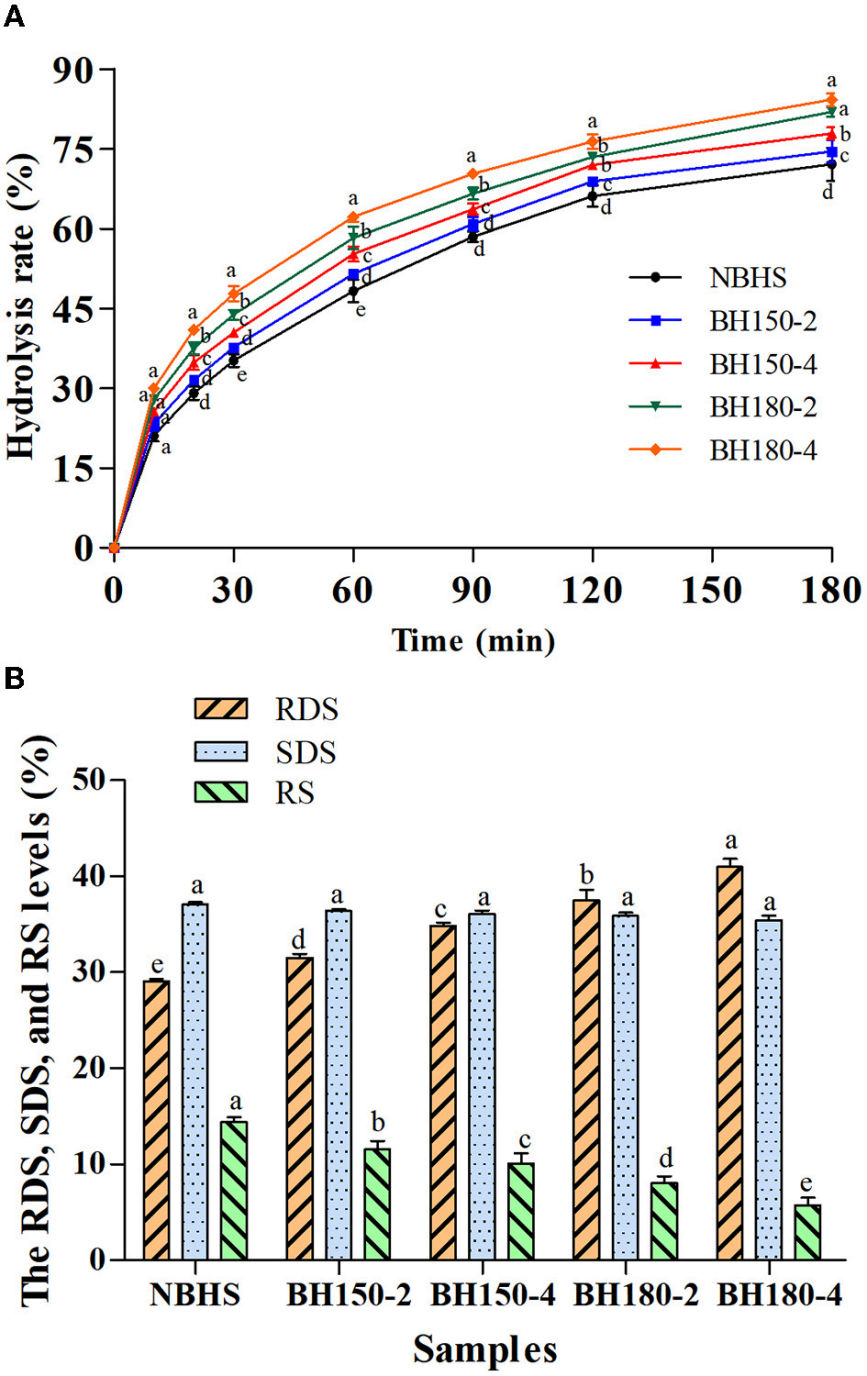
Hydrolysis rate **(A)** and RDS, SDS, and RS levels **(B)** of different samples. Bars bearing different letters in the same time point **(A)** and different samples **(B)** within the same property are significantly different (*p* < 0.05).

The effect of DHT on *in vitro* digestibility of starches is shown in Figure 6A, and the level of RDS, SDS, and RS is presented in Figure 6B. Total hydrolysis of all samples increased as the digestion time prolonged, in the first 30 min of which, it increased steeply, and then gradually from 30 to 180 min. Compared with NBHS, the RDS content of DHT-modified starches increased, reaching a maximum value at BH180-4 (11.90%), while the content of SDS and RS decreased (Figure 6B). These results suggested that the *in vitro* digestibility of BH starch had been improved after DHT. Similar trends had been previously observed on waxy potato and quinoa starches following DHT (52).

After DHT, the AMC of modified starches significantly decreased, making them more susceptible to enzymatic digestion (53). This might be one factor for the RDS content increasing. As shown by DSC results (gelatinization temperatures and Δ*H* decrease), the disruption of double helices in the crystalline region would also cause an increase in RDS content (44). The partial disruption or damage of organized starch chains and weak associations between starch molecules would increase the susceptibility of BH starch to enzymatic digestion concurrently responsible for the decreased RS content (33). Furthermore, the high temperature and low moisture content during DHT impaired starch granules to form a porous structure. Thus, it was prone to enzyme attack and inclined to draw digestive enzymes into the interior of starch granules (53). The transition from RS to RDS during DHT might account for these results as well.

### 3.12. PCA of structural, physicochemical, and digestive properties

**Figure 7:**
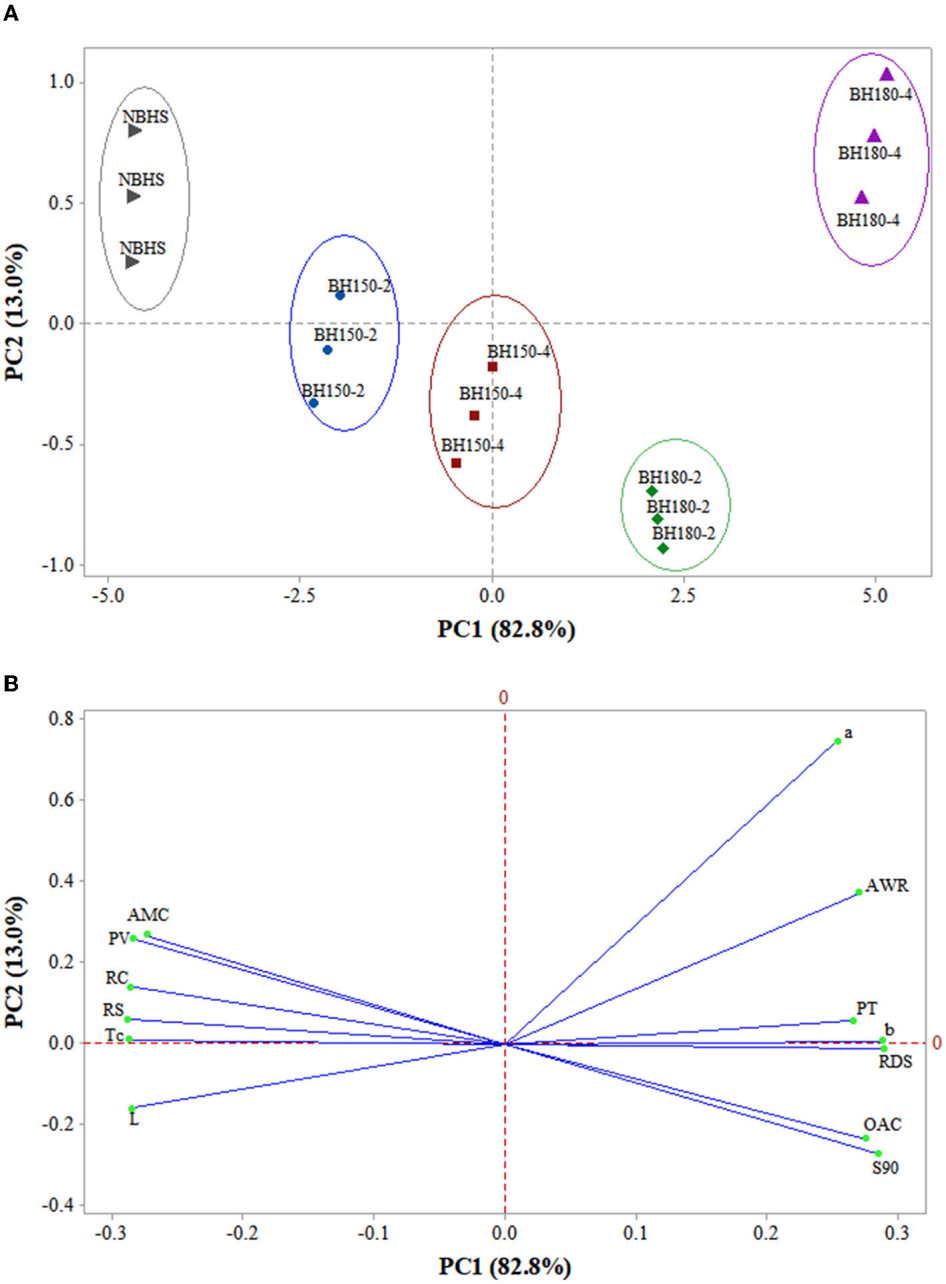
PCA biplots summarizing the relationships between samples and their microstructural, physicochemical, and digestibility properties. **(A)** The clusters of different samples on the score plot; **(B)** PCA loading plot of different properties. L, the lightness of samples; a, red/green axis of color analysis; b, yellow/blue axis of color analysis; PT, pasting temperature; PV, peak viscosity; Tc, concluding temperature; AMC, amylose content; RC, relative crystallinity; AWR, alkaline water retention; OAC, oil absorption capacity; S90, solubility at 90°C; RDS, rapidly digestible starch; RS, resistant starch.

The PCA of structural, physicochemical, and digestive properties among NBHS, BH150-2, BH150-4, BH180-2, and BH180-4 is shown in Figure 7. The PC1 and PC2, respectively, represented 82.8 and 13.0% of the total variance, totaling 95.8%. As shown in the score plot (Figure 7A), NBHS, BH150-2, and BH15-4 were at the negative part of PC1, while BH180-2 and BH180-4 were at the positive part of PC1. In addition, NBHS, BH150-4, BH180-2, and BH180-4 were located at two different sides of PC2, respectively; BH150-2 was nearly located on the PC2 line. The distance between any two samples positively correlated with the degree of differences between them. The distance between BH150-2 and BH150-4 was relatively close, suggesting that their structural, physicochemical, and digestive properties were similar. However, the BH150-2, BH150-4, BH180-2, and BH180-4 gradually moved farther from NBHS. This indicated that DHT surely had various effects on the structural, physicochemical, and digestive properties of BH starch. The degree of effect was positively related to the treatment temperature and duration, in which case BH180-4 was altered to the greatest extent.

Furthermore, AMC, PV, RC, RS, T_c_, and L stayed at the negative part of PC1 on the loading plot (Figure 7B) and were close to NBHS and BH150-2 on the score plot, indicating they were highly correlated. a, AWR, PT, b, RDS, OAC, and S90 were at the positive part of PC1 and positively related to BH180-2 and BH180-4. These results indicated that the DHT had huge effects on the structure, AMC, thermostability, physicochemical, and digestive functions of BH starch. Therefore, PCA established a strong relationship mainly among structural, physicochemical, and digestive characteristics, and showed some clusters of different samples.

## 4. Conclusion

Although Blue highland barley (BH) is rich in dietary nutrients, its health-promoting potential is yet to be explored. In this study, DHT, as a safe and maneuverable physical method for starch modification with limited by-products, was introduced to modify BH starch. The effect on structural, physicochemical, and digestive properties of BH starch showed a thermodynamic impact with heating temperature and duration variations. The DHT greatly increased potholes and fissures on the surface of BH starch, while decreasing the AMC, RC, and R_1, 047/1, 022_. The AWR, water and oil absorption capacities, solubility, and LT significantly increased after DHT, whereas the SP, gelatinization characteristics, and viscosities decreased. All these changes were positively correlated with DHT temperature and duration. In addition, *in vitro* hydrolysis rate of BH starch was remarkably improved by DHT. The RDS content of modified samples displayed an upward trend along with temperature and duration increase; however, the RS content decreased significantly. Overall, the results identified that DHT effectively altered the structures, physicochemical properties, and *in vitro* digestion of BH starch. The improvements in oil absorption capacity, viscosities, and gelatinization properties after DHT will promote BH starch in food industrial applications. DHT-modified BH starch might be utilized in functional food development by interacting with dietary polyphenols and lipids in the future. In addition, this manuscript provided more theoretical information about DHT on starch modification.

## Data availability statement

The original contributions presented in the study are included in the article/supplementary material, further inquiries can be directed to the corresponding author.

## Author contributions

SL: conceptualization, investigation, methodology, formal analysis, validation, and writing-original draft. HL: supervision, formal analysis, writing-review and editing, and funding acquisition. SG: writing-review and editing and data curation. SsG: methodology. CZ: resources and funding management. All authors contributed to the article and approved the submitted version.
